# Imaging opens possibilities both to target and to evaluate nutrition in critical illness

**DOI:** 10.1186/cc13882

**Published:** 2014-05-21

**Authors:** Olav Rooyackers, Jan Wernerman

**Affiliations:** 1 Department of Anesthesia and Intensive Care Medicine, K32, Karolinska University Hospital Huddinge, 14186 Stockholm, Sweden

## 

Recently, a number of clinical trials have investigated the effect of nutrition in critical illness [1-4]. The results have been confusing rather than clarifying. Obviously there are difficulties in defining whether or not a specific patient is at a nutritional risk and also what should be the nutritional target in the early course of critical illness. The step from a nutritional intervention in the ICU to mortality outcomes is just too big and there is no well-known underlying mechanism. Not surprisingly, there are no prospective randomised studies demonstrating survival advantages in relation to nutrition.

Loss of muscle mass is a cardinal symptom in patients with need for a prolonged ICU stay. Degree of muscle depletion has been demonstrated to relate to success of rehabilitation efforts as well as to post-ICU quality of life. But the actual loss of protein in muscle must be quantified in a biopsy specimen [5-7], which makes it very difficult to use protein content as a marker in the individual subject in clinical practice (Figure 1). In addition, there is no direct relation between muscle mass and muscle function. Tests of muscle function during critical illness are difficult because tests independent of patient compliance are needed. Measures of muscle turnover are often invasive and therefore are limited in terms of repeats. Invasive measuring will still be necessary to clarify underlying mechanisms, but will never be a part of clinical practice. Imaging is therefore very attractive, particularly if imaging devices can be utilised at the bedside.

**Figure 1:**
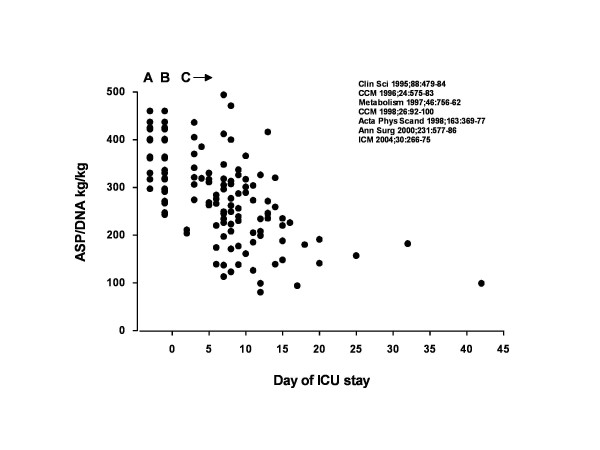
**Protein content in skeletal muscle related to DNA.** Protein content in skeletal muscle (ASP – Alkali Soluble Protein) related to DNA in **(A)** healthy subjects, **(B)** preoperative patients undergoing elective surgery, and **(C)** critically ill patients (*n* = 147). Data assembled from a number of published studies by the authors as indicated [5,6,8-12].

Abdominal computed tomography scans, performed for other purposes, have been utilised in oncological patients to diagnose sarcopenia and to demonstrate the progress of sarcopenia over time [13,14]. This concept is now transferred to critically ill patients in the ICU [15,16], and in a recent report Weijs and colleagues reported a predictive value of sarcopenia during the ICU stay [17]. In a subgroup within the Early Parenteral Nutrition Completing Enteral Nutrition in Adult Critically Ill Patients study, computed tomography scanning of the leg and abdomen was also used to assess the temporal loss of muscle tissue [18]. Within that study the losses of leg muscle in repeated measurements were demonstrated to be useful to evaluate a nutritional intervention. The possibility to include objective measurements related to muscle wasting that are possible to handle in everyday clinical practice is an advancement in critical care medicine that should not be underestimated.

The limitation in using computed tomography scans in clinical practice today is that it involves patient transportation, which may involve medical risks and use of resources. Another track involving imaging and estimation of skeletal muscle mass, which easily can be brought to the bedside, is the use of ultrasound. Several investigators have demonstrated the use of ultrasound to monitor sarcopenia in critically ill patients over time [19]. In a recent study, ICU patients were investigated with repeated ultrasound assessment of leg muscle [20]. A relation between the severity of illness and the extent of the shrinkage of muscle cross-sectional area was reported. In addition, the report included biochemical data for protein content and protein kinetics in subgroups of patients in pllel to the imaging data. Although the report did not include any linking of the imaging by ultrasound to the biochemical alterations in skeletal muscle, which reflects the mechanisms behind the rather aggressive muscle depletion often seen in critical illness, the effort to present measurements obtainable on an everyday clinical basis to underlying mechanistic data is a conceptual breakthrough.

The recent developments of imaging and tissue signalling should in the near future make it possible to define changes over time in individual patients and to link this to clinically relevant correlates. This future is particularly true for the measures of gene expression and signalling [21,22]. This link is well demonstrated in critically ill patients in relation to skeletal muscle degradation [22-24], which is the major mechanistic component of the muscle depletion seen (Figure 2). Overall, the changes seen in gene expression and signalling in skeletal muscle during critical illness should be linked both to physiological changes in protein kinetics and to clinical relevant data [22,25]. Here the imaging may prove to be helpful. It seems plausible to predict a development where individual changes in estimates of muscle mass will be sufficiently accurate to be directly linked to the quantitative measures of underlying mechanisms. The data presented by Casaer and colleagues indicate this type of precision when changes in muscle volume were statistically related to changes in body weight and caloric intake [18].

**Figure 2:**
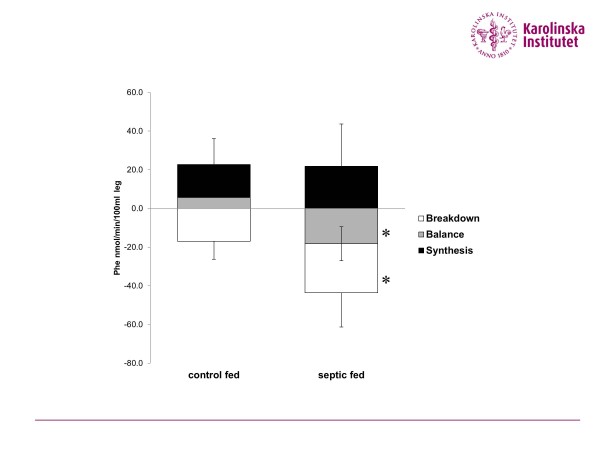
**Muscle protein turnover in fed critically ill patients and healthy volunteers.** Muscle protein turnover data from fed critically ill patients (*n* = 7) and fed healthy volunteers (*n* = 8). Both groups were fed according to energy expenditure. Muscle protein turnover was measured and calculated using isotopically labelled phenylalanine (Phe) and employing a two-pool model of the leg. Bars demonstrate a similar muscle protein synthesis rate, but a different protein degradation rate (*P* < 0.05), resulting in a different protein balance (*P* < 0.05). Data illustrate the underlying mechanism behind the rapid development of sarcopenia in critical illness. Data with permission from [22]. *Statistically different from healthy controls, *P* < 0.05.

Today there are every few studies in which post-ICU muscle function is evaluated. Interesting pilot studies are at hand [26], but the limitation is often the difficulty to recruit a representative sample. There is an evolving literature over post-ICU quality of life in general, usually relying upon questionnaires that are well validated [27,28]. Again, the limitation is most often the difficulty to recruit representative samples. Nevertheless, awareness of the difficulties involved means that the accuracy of results is improving over time. Imaging may be helpful in bridging the clinical finding during the ICU stay to functional status post-ICU. The link between sarcopenia during the ICU stay as a possible reflection of malnutrition and mortality outcome presented by Weijs and colleagues is a promising example in this direction [17].

## Competing interests

The authors declare that they have no competing interests.
